# [1–9-NαC]-Linusorb B3 (Cyclo­linopeptide A) dimethyl sulfoxide monosolvate

**DOI:** 10.1107/S2414314620003181

**Published:** 2020-03-13

**Authors:** Sarah Kendra Purdy, Denis Spasyuk, Jackson Mulenga Chitanda, Martin J. T. Reaney

**Affiliations:** a Department of Plant Sciences, College of Agriculture and Bioresources, University of Saskatchewan, 51 Campus Dr., Saskatoon, Saskatchewan, S7N 5A8, Canada; b Canadian Light Source Inc., 44 Innovation Blvd., Saskatoon, Saskatchewan, S7N 3V4, Canada; c Guangdong Saskatchewan Oilseed Joint Laboratory, Department of Food Science and Engineering, Jinan University, 601 Huangpu Avenue West, Guangdong, Guangzhou, 510632, People’s Republic of China; University of Toronto, Canada

**Keywords:** crystal structure, cyclic peptide, orbitides, cyclo­linopeptide A, [1–9-NαC]-linusorb B3, dimethyl sulfoxide, hydrogen bonding

## Abstract

Crystals of the dimethyl sulfoxide (DMSO) solvate of [1–9-NαC]-linusorb B3 (Cyclo­linopeptide A; CLP-A; C_57_H_84_N_9_O_9_·C_2_H_6_OS), a cyclic polypeptide were obtained following peptide extraction and purification from flaxseed oil. There are four intramolecular N—H⋯O hydrogen bonds. In the crystal, the mol­ecules are linked in chains along the *a* axis by N—H⋯O hydrogen bonds. Each DMSO O atom accepts a hydrogen bond from an NH group at the Phe^6^ location in the CLP-A mol­ecule.

## Abstract 2

[1–9-NαC]-Linusorb B3 (Cyclo­linopeptide A) was extracted from flaxseed oil crystals formed in dimethyl sulfoxide. The mol­ecule has four intra­molecular N—H⋯O hydrogen bonds, and the DMSO solvate mol­ecule is bound to the Phe^6^ amino acid by a fifth N—H⋯O hydrogen bond.

## Graphical abstract

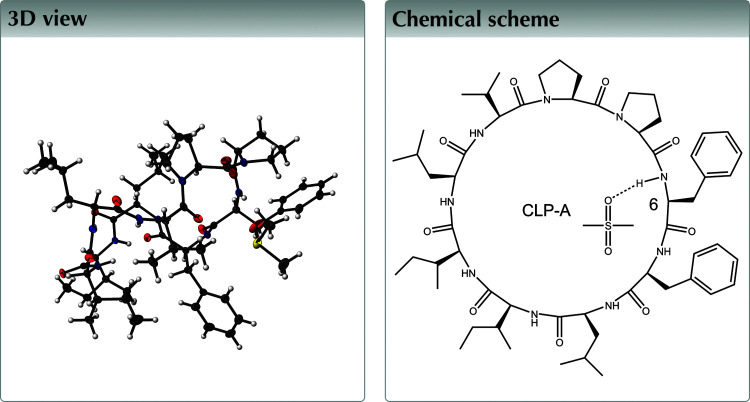

## Structure description

**Figure 1:**
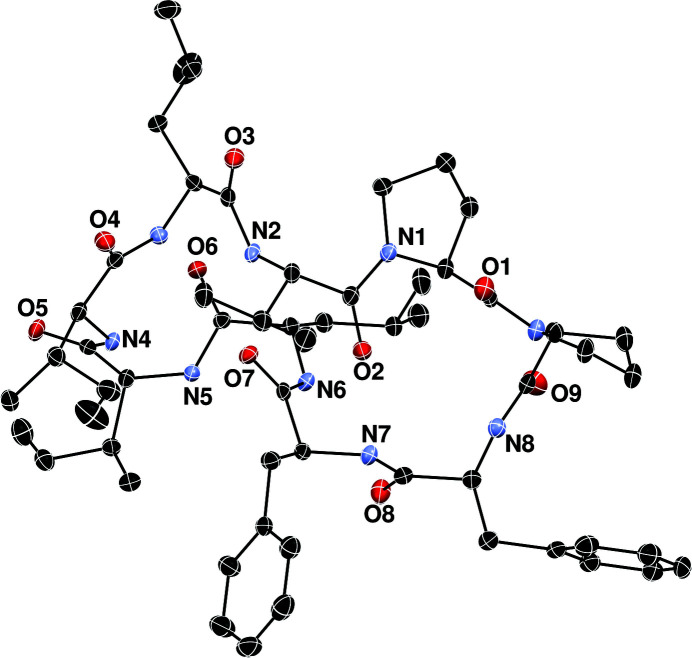
The mol­ecular structure of the title compound. Displacement ellipsoids are drawn at 50% probability level. The DMSO solvent mol­ecule is not shown.

**Figure 2:**
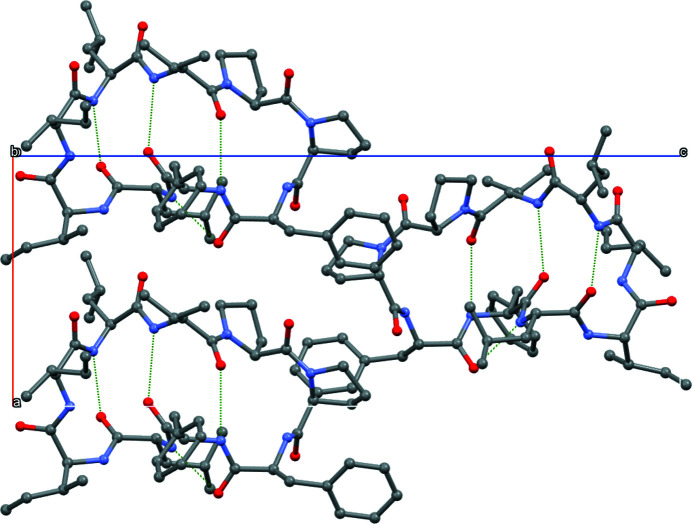
View along the *b* axis showing crystal packing of the title compound. The hydrogen bonds are shown as dashed lines and H atoms have been omitted for clarity.

The mol­ecular structure of the title compound is shown in Fig. 1 ▸. The cyclic polypeptide or orbitide [1–9-NαC]-linusorb B3 (Cyclo­linopeptide A; CLP-A) has nine amino acids (Ile1–Leu2–Val3–Pro4–Pro5–Phe6–Phe7–Leu8–Ile9). The nomenclature and amino-acid numbering for orbitides was standardized by Craik *et al.* (2016 ▸). The title compound was first isolated from flax seed by Kaufmann & Tobschirbel (1959 ▸). The current method involves the use of silica gel chromatography to extract cyclic peptides from unrefined flaxseed oil, followed by isolation of the orbitide using high performance liquid chromatography (Reaney *et al.*, 2013 ▸). The isolated orbitide was then dissolved in dimethyl sulfoxide (DMSO) and stored under ambient conditions. The mol­ecule has four intra­molecular N—H⋯O hydrogen bonds, and the DMSO solvate mol­ecule is bound to the Phe6 amino acid by an N—H⋯O hydrogen bond (Table 1 ▸). The packing is shown in Fig. 2 ▸. The first crystal structure for CLP-A 2-propanol solvate was published by Di Blasio *et al.*, 1989 ▸), followed by the same compound in methanol/2-propanol (Matsumoto *et al.*, 2002 ▸), methanol (Quail *et al.*, 2009 ▸), and in aceto­nitrile (Chitanda *et al.*, 2016 ▸).

The compound, [1–9-NαC]-linusorb B3, has shown to induce potentially beneficial responses in living organisms. The biomolecular inter­action with human albumin has been reported by Rempel *et al.* (2010 ▸). It has demonstrated cytoprotective activity in liver cells by inhibiting cholate uptake (Kessler *et al.*, 1986 ▸). The title compound has been shown to have immunosuppressive activity, and no toxicity at high doses (Wieczorek *et al.*, 1991 ▸; Gaymes *et al.*, 1997 ▸).

## Synthesis and crystallization

The crystals were found unintentionally after the title compound was dissolved in DMSO, and allowed to evaporate slowly at ambient temperature. Single crystal X-ray diffraction data for the title compound were collected using the Canadian Macromolecular Crystallography Facility CMCF-BM beamline at the Canadian Light Source (CLS), described by Grochulski *et al.* (2011 ▸). The CMCF-BM is a bending magnet beamline equipped with an Si (111) double-crystal monochromator, Rayonix MX300HE CCD detector and MD2 microdiffractometer equipped with Mini Kappa Goniometer Head. Data for the title compound were collected at 18.000 keV (0.68882 Å) and 100 K.

## Refinement

Crystal data, data collection and structure refinement details are summarized in Table 2 ▸. Cell refinement and data reduction were performed using *XDS* (Kabsch, 1993 ▸). A semi-empirical absorption correction, based on the multiple measurements of equivalent reflections, and merging of data was performed using *SADABS* (Krause *et al.*, 2015 ▸). Data conversion from *XDS* file format to *SADABS* file format was performed using *XDS2SAD* (Sheldrick, 2008*a* ▸). The space group was confirmed by *XPREP* routines (Bruker, 2014 ▸). The structures were solved by direct-methods and refined by full-matrix least squares and difference-Fourier techniques with *SHELXL2016* (Sheldrick, 2015 ▸). All non-H atoms were refined by full-matrix least squares with anisotropic displacement parameters. A final verification of possible voids was performed using the VOID routine of *PLATON* (Spek, 2020 ▸). The *checkCIF* routine and structure-factor analyses were performed by *PLATON* (Spek, 2020 ▸). All publication materials were prepared using *LinXTL* (Spasyuk, 2009 ▸) and *Mercury* (Macrae *et al.*, 2020 ▸).

## Supplementary Material

Crystal structure: contains datablock(s) I, global. DOI: 10.1107/S2414314620003181/lh4052sup1.cif

Structure factors: contains datablock(s) I. DOI: 10.1107/S2414314620003181/lh4052Isup2.hkl

Supporting information file. DOI: 10.1107/S2414314620003181/lh4052Isup3.cml

CCDC reference: 1988784

Additional supporting information:  crystallographic information; 3D view; checkCIF report

## Figures and Tables

**Table 1:** Hydrogen-bond geometry (Å, °)

*D*—H⋯*A*	*D*—H	H⋯*A*	*D*⋯*A*	*D*—H⋯*A*
N2—H2*N*⋯O7	0.88	2.12	2.970 (3)	162
N3—H3*N*⋯O6	0.88	2.16	3.023 (3)	166
N4—H4*N*⋯O7	0.88	2.29	3.117 (3)	157
N5—H5*N*⋯O3^i^	0.88	2.38	3.094 (3)	139
N6—H6*N*⋯O8	0.88	2.16	2.926 (3)	145
N8—H8*N*⋯O1*S*	0.88	2.05	2.800 (3)	150

Symmetry code: (i) 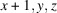 .

**Table 2:** Experimental details

Crystal data	
Chemical formula	C_57_H_84_N_9_O_9_·C_2_H_6_OS
*M*_r_	1117.45
Crystal system, space group	Orthorhombic, *P*2_1_2_1_2_1_
Temperature (K)	100
*a*, *b*, *c* (Å)	9.942 (2), 22.986 (5), 26.512 (5)
*V* (Å^3^)	6059 (2)
*Z*	4
Radiation type	Synchrotron, λ = 0.68882 Å
μ (mm^−1^)	0.11
Crystal size (mm)	0.05 × 0.01 × 0.01

Data collection	
Diffractometer	Rayonix MX300HE CCD area detector
Absorption correction	Multi-scan (*SADABS*; Sheldrick, 1996)
*T*_min_, *T*_max_	0.415, 0.494
No. of measured, independent and observed [*I* > 2σ(*I*)] reflections	83438, 11739, 10684
*R*_int_	0.083
(sin θ/λ)_max_ (Å^−1^)	0.614

Refinement	
*R*[*F* ^2^ > 2σ(*F* ^2^)], *wR*(*F* ^2^), *S*	0.039, 0.101, 1.04
No. of reflections	11739
No. of parameters	726
No. of restraints	6
H-atom treatment	H-atom parameters constrained
Δρ_max_, Δρ_min_ (e Å^−3^)	0.39, −0.30
Absolute structure	Flack *x* determined using 4488 quotients [(*I* ^+^)−(*I* ^−^)]/[(*I* ^+^)+(*I* ^−^)] (Parsons *et al.*, 2013 ▸)
Absolute structure parameter	0.00 (4)

Computer programs: *MxDC* (Fodje, 2012 ▸), *XDS* (Kabsch, 2010 ▸), *SHELXS* (Sheldrick, 2008*b* ▸), *SHELXL2016* (Sheldrick, 2015 ▸), *ShelXle* (Hübschle *et al.*, 2011 ▸), *LinXTL* (Spasyuk, 2009 ▸) and *Mercury* (Macrae *et al.*, 2020 ▸).

## full crystallographic data

### Crystal data

**Table:**

C_57_H_84_N_9_O_9_·C_2_H_6_OS	*D*_x_ = 1.225 Mg m^−^^3^
*M**_r_* = 1117.45	Synchrotron radiation, λ = 0.68882 Å
Orthorhombic, *P*2_1_2_1_2_1_	Cell parameters from 17346 reflections
*a* = 9.942 (2) Å	θ = 1.1–30.5°
*b* = 22.986 (5) Å	µ = 0.11 mm^−^^1^
*c* = 26.512 (5) Å	*T* = 100 K
*V* = 6059 (2) Å^3^	Needle, colourless
*Z* = 4	0.05 × 0.01 × 0.01 mm
*F*(000) = 2412	

### Data collection

**Table:**

Rayonix MX300HE CCD area detector diffractometer	10684 reflections with *I* > 2σ(*I*)
Radiation source: synchrotron	*R*_int_ = 0.083
κ and ω scans	θ_max_ = 25.0°, θ_min_ = 1.1°
Absorption correction: multi-scan (SADABS; Sheldrick, 1996)	*h* = −12→12
*T*_min_ = 0.415, *T*_max_ = 0.494	*k* = −28→28
83438 measured reflections	*l* = −32→32
11739 independent reflections	

### Refinement

**Table:**

Refinement on *F*^2^	H-atom parameters constrained
Least-squares matrix: full	*w* = 1/[σ^2^(*F*_o_^2^) + (0.0567*P*)^2^ + 1.3012*P*] where *P* = (*F*_o_^2^ + 2*F*_c_^2^)/3
*R*[*F*^2^ > 2σ(*F*^2^)] = 0.039	(Δ/σ)_max_ = 0.001
*wR*(*F*^2^) = 0.101	Δρ_max_ = 0.39 e Å^−^^3^
*S* = 1.04	Δρ_min_ = −0.29 e Å^−^^3^
11739 reflections	Extinction correction: SHELXL2016 (Sheldrick, 2015), Fc^*^=kFc[1+0.001xFc^2^λ^3^/sin(2θ)]^-1/4^
726 parameters	Extinction coefficient: 0.0137 (8)
6 restraints	Absolute structure: Flack *x* determined using 4488 quotients [(*I*^+^)-(*I*^-^)]/[(*I*^+^)+(*I*^-^)] (Parsons *et al.*, 2013)
Hydrogen site location: inferred from neighbouring sites	Absolute structure parameter: 0.00 (4)

### Special details

**Table:**

Geometry. All esds (except the esd in the dihedral angle between two l.s. planes) are estimated using the full covariance matrix. The cell esds are taken into account individually in the estimation of esds in distances, angles and torsion angles; correlations between esds in cell parameters are only used when they are defined by crystal symmetry. An approximate (isotropic) treatment of cell esds is used for estimating esds involving l.s. planes.
Refinement. The H atoms were generated geometrically (C—H 0.93 to 0.98, N—H 0.86 and O—H 0.82?Å) and were included in the refinement in the riding model approximation; their displacement parameterwere set to 1.5 times those of the equivalent isotropic temperature factors of the parent site (methyl) and 1.2 times for others.

### Fractional atomic coordinates and isotropic or equivalent isotropic displacement parameters (Å^2^)

**Table:**

	*x*	*y*	*z*	*U*_iso_*/*U*_eq_	
C1	0.2960 (3)	0.28363 (12)	0.63624 (9)	0.0186 (5)	
H1	0.393302	0.282822	0.645792	0.022*	
C2	0.2427 (3)	0.22180 (12)	0.62660 (10)	0.0237 (6)	
H2A	0.252439	0.210891	0.590659	0.028*	
H2B	0.290573	0.192908	0.647690	0.028*	
C3	0.0946 (3)	0.22615 (13)	0.64156 (10)	0.0251 (6)	
H3A	0.040534	0.243691	0.614134	0.030*	
H3B	0.057325	0.187372	0.649831	0.030*	
C4	0.0969 (3)	0.26534 (12)	0.68789 (10)	0.0206 (5)	
H4A	0.012561	0.288029	0.690720	0.025*	
H4B	0.109675	0.242353	0.719097	0.025*	
C5	0.2395 (3)	0.35445 (12)	0.70124 (9)	0.0187 (5)	
C6	0.1467 (3)	0.37058 (12)	0.74561 (9)	0.0188 (5)	
H6	0.052590	0.359539	0.736289	0.023*	
C7	0.1481 (3)	0.43575 (12)	0.75826 (10)	0.0223 (6)	
H7	0.241919	0.447526	0.767243	0.027*	
C8	0.1014 (3)	0.47150 (13)	0.71281 (11)	0.0314 (7)	
H8A	0.009380	0.460273	0.703821	0.047*	
H8B	0.161304	0.464148	0.684159	0.047*	
H8C	0.103614	0.512966	0.721363	0.047*	
C9	0.0561 (3)	0.44747 (14)	0.80322 (10)	0.0260 (6)	
H9A	−0.036005	0.435887	0.794761	0.039*	
H9B	0.058022	0.489042	0.811388	0.039*	
H9C	0.087348	0.425008	0.832376	0.039*	
C10	0.1040 (3)	0.29885 (12)	0.81360 (9)	0.0196 (5)	
C11	0.1734 (3)	0.25834 (12)	0.85275 (9)	0.0189 (5)	
H11	0.218667	0.227067	0.832770	0.023*	
C12	0.0714 (3)	0.22740 (12)	0.88682 (10)	0.0217 (6)	
H12A	0.108189	0.225277	0.921469	0.026*	
H12B	−0.012287	0.250715	0.888119	0.026*	
C13	0.0374 (3)	0.16610 (12)	0.86894 (10)	0.0229 (6)	
H13	0.032229	0.166534	0.831270	0.027*	
C14	−0.0994 (3)	0.14716 (13)	0.88945 (12)	0.0309 (7)	
H14A	−0.095534	0.145196	0.926342	0.046*	
H14B	−0.122357	0.108758	0.875900	0.046*	
H14C	−0.168082	0.175400	0.879249	0.046*	
C15	0.1460 (4)	0.12289 (15)	0.88447 (13)	0.0405 (8)	
H15A	0.233709	0.136566	0.872430	0.061*	
H15B	0.126423	0.084763	0.869642	0.061*	
H15C	0.147853	0.119545	0.921310	0.061*	
C16	0.2541 (3)	0.33778 (11)	0.90634 (9)	0.0175 (5)	
C17	0.3714 (3)	0.36376 (11)	0.93570 (9)	0.0173 (5)	
H17	0.367420	0.346928	0.970449	0.021*	
C18	0.3572 (3)	0.42997 (11)	0.94141 (10)	0.0197 (5)	
H18	0.265542	0.437952	0.955322	0.024*	
C19	0.3683 (3)	0.46159 (12)	0.89083 (11)	0.0264 (6)	
H19A	0.464486	0.466299	0.882138	0.032*	
H19B	0.326086	0.437382	0.864300	0.032*	
C20	0.3014 (4)	0.52111 (14)	0.89121 (15)	0.0450 (9)	
H20A	0.204173	0.516447	0.895660	0.067*	
H20B	0.319219	0.540921	0.859161	0.067*	
H20C	0.337842	0.544324	0.919046	0.067*	
C21	0.4601 (3)	0.45288 (12)	0.97960 (10)	0.0237 (6)	
H21A	0.447175	0.433178	1.012017	0.036*	
H21B	0.447631	0.494863	0.984028	0.036*	
H21C	0.551206	0.445247	0.967159	0.036*	
C22	0.5943 (3)	0.31953 (11)	0.94307 (9)	0.0159 (5)	
C23	0.7263 (3)	0.30260 (11)	0.91749 (9)	0.0161 (5)	
H23	0.761008	0.266614	0.934216	0.019*	
C24	0.8330 (3)	0.35108 (11)	0.92385 (9)	0.0179 (5)	
H24	0.917834	0.336121	0.908276	0.021*	
C25	0.8658 (3)	0.36478 (12)	0.97918 (10)	0.0227 (6)	
H25A	0.936220	0.395207	0.980096	0.027*	
H25B	0.784329	0.381011	0.995399	0.027*	
C26	0.9137 (3)	0.31302 (14)	1.00990 (11)	0.0308 (7)	
H26A	0.839559	0.285303	1.014003	0.046*	
H26B	0.943818	0.326345	1.043138	0.046*	
H26C	0.988494	0.293997	0.992380	0.046*	
C27	0.7970 (3)	0.40725 (12)	0.89575 (10)	0.0219 (6)	
H27A	0.719429	0.425657	0.912033	0.033*	
H27B	0.774733	0.398084	0.860632	0.033*	
H27C	0.873892	0.433915	0.896630	0.033*	
C28	0.6247 (3)	0.25351 (11)	0.84263 (9)	0.0156 (5)	
C29	0.6293 (3)	0.24907 (11)	0.78470 (9)	0.0166 (5)	
H29	0.537432	0.237960	0.772717	0.020*	
C30	0.7264 (3)	0.20104 (11)	0.76897 (9)	0.0188 (5)	
H30A	0.697160	0.164277	0.785029	0.023*	
H30B	0.816588	0.210573	0.782483	0.023*	
C31	0.7395 (3)	0.19054 (12)	0.71180 (10)	0.0210 (6)	
H31	0.784709	0.225132	0.696444	0.025*	
C32	0.8294 (3)	0.13762 (13)	0.70376 (11)	0.0291 (7)	
H32A	0.917224	0.144533	0.719472	0.044*	
H32B	0.841472	0.130952	0.667529	0.044*	
H32C	0.787444	0.103349	0.719108	0.044*	
C33	0.6040 (3)	0.18198 (14)	0.68568 (11)	0.0299 (7)	
H33A	0.618502	0.175361	0.649566	0.045*	
H33B	0.548582	0.216823	0.690358	0.045*	
H33C	0.558023	0.148281	0.700340	0.045*	
C34	0.5851 (3)	0.35148 (11)	0.76922 (9)	0.0153 (5)	
C35	0.6307 (3)	0.40770 (11)	0.74322 (9)	0.0157 (5)	
H35	0.723693	0.416526	0.755347	0.019*	
C36	0.5415 (3)	0.45916 (11)	0.75762 (10)	0.0192 (5)	
H36A	0.461146	0.459114	0.735553	0.023*	
H36B	0.510184	0.453725	0.792753	0.023*	
C37	0.6096 (3)	0.51789 (11)	0.75357 (9)	0.0186 (5)	
C38	0.7203 (3)	0.53083 (12)	0.78370 (11)	0.0256 (6)	
H38	0.754196	0.502397	0.806383	0.031*	
C39	0.7816 (3)	0.58518 (14)	0.78079 (13)	0.0337 (7)	
H39	0.857698	0.593563	0.801211	0.040*	
C40	0.7316 (4)	0.62720 (14)	0.74804 (12)	0.0350 (8)	
H40	0.773500	0.664284	0.746013	0.042*	
C41	0.6213 (4)	0.61485 (13)	0.71858 (11)	0.0325 (7)	
H41	0.586584	0.643479	0.696251	0.039*	
C42	0.5607 (3)	0.56058 (12)	0.72150 (11)	0.0244 (6)	
H42	0.484365	0.552515	0.701133	0.029*	
C43	0.7495 (3)	0.37703 (11)	0.66673 (9)	0.0171 (5)	
C44	0.7523 (3)	0.37092 (11)	0.60924 (10)	0.0176 (5)	
H44	0.821366	0.340737	0.601048	0.021*	
C45	0.7996 (3)	0.42808 (12)	0.58501 (10)	0.0218 (6)	
H45A	0.732658	0.458919	0.592022	0.026*	
H45B	0.885902	0.439987	0.600538	0.026*	
C46	0.8183 (3)	0.42259 (11)	0.52855 (10)	0.0205 (6)	
C47	0.7286 (3)	0.44815 (12)	0.49512 (11)	0.0258 (6)	
H47	0.654132	0.469597	0.507672	0.031*	
C48	0.7473 (3)	0.44256 (14)	0.44313 (11)	0.0304 (7)	
H48	0.684951	0.459984	0.420550	0.036*	
C49	0.8557 (4)	0.41183 (13)	0.42429 (10)	0.0299 (7)	
H49	0.868752	0.408569	0.388907	0.036*	
C50	0.9456 (3)	0.38574 (12)	0.45745 (11)	0.0264 (6)	
H50	1.019783	0.364229	0.444697	0.032*	
C51	0.9273 (3)	0.39105 (12)	0.50941 (10)	0.0215 (6)	
H51	0.989108	0.373159	0.531908	0.026*	
C52	0.6105 (3)	0.29527 (11)	0.57304 (9)	0.0189 (5)	
C53	0.4766 (3)	0.27766 (11)	0.54925 (10)	0.0194 (5)	
H53	0.446135	0.240040	0.564345	0.023*	
C54	0.4909 (3)	0.27020 (13)	0.49163 (10)	0.0260 (6)	
H54A	0.583710	0.258824	0.482363	0.031*	
H54B	0.427386	0.240571	0.478695	0.031*	
C55	0.4568 (3)	0.33019 (13)	0.47142 (10)	0.0269 (6)	
H55A	0.429480	0.328207	0.435557	0.032*	
H55B	0.534519	0.356876	0.474636	0.032*	
C56	0.3405 (3)	0.34999 (13)	0.50447 (9)	0.0246 (6)	
H56A	0.340378	0.392809	0.508530	0.029*	
H56B	0.253080	0.337572	0.490196	0.029*	
C57	0.2729 (3)	0.32246 (12)	0.59002 (9)	0.0186 (5)	
N1	0.2127 (2)	0.30369 (10)	0.67852 (8)	0.0182 (5)	
N2	0.1890 (2)	0.33375 (10)	0.78791 (8)	0.0195 (5)	
H2N	0.274079	0.334440	0.796999	0.023*	
N3	0.2804 (2)	0.28902 (9)	0.87956 (8)	0.0175 (4)	
H3N	0.363179	0.275563	0.878257	0.021*	
N4	0.5000 (2)	0.34647 (9)	0.91426 (8)	0.0156 (4)	
H4N	0.516972	0.353552	0.882265	0.019*	
N5	0.7134 (2)	0.29074 (9)	0.86333 (8)	0.0162 (4)	
H5N	0.767876	0.309418	0.842783	0.019*	
N6	0.6638 (2)	0.30463 (10)	0.76177 (8)	0.0172 (4)	
H6N	0.736352	0.307591	0.742953	0.021*	
N7	0.6377 (2)	0.39981 (9)	0.68821 (8)	0.0171 (4)	
N8	0.6252 (2)	0.35076 (9)	0.58825 (8)	0.0177 (4)	
H8N	0.557278	0.375122	0.585489	0.021*	
N9	0.3678 (2)	0.32044 (10)	0.55315 (8)	0.0191 (5)	
O1	0.17144 (19)	0.35242 (9)	0.58599 (7)	0.0232 (4)	
O2	0.33654 (19)	0.38488 (9)	0.68831 (7)	0.0238 (4)	
O3	−0.01718 (19)	0.29596 (9)	0.80507 (7)	0.0246 (4)	
O4	0.14142 (19)	0.35982 (8)	0.90759 (7)	0.0224 (4)	
O5	0.58021 (19)	0.31123 (9)	0.98829 (6)	0.0217 (4)	
O6	0.5431 (2)	0.22437 (8)	0.86719 (7)	0.0200 (4)	
O7	0.48473 (18)	0.34897 (8)	0.79686 (6)	0.0186 (4)	
O8	0.84805 (19)	0.36238 (9)	0.69175 (7)	0.0248 (4)	
O9	0.6992 (2)	0.25795 (9)	0.57647 (8)	0.0298 (5)	
O1S	0.4825 (2)	0.45567 (10)	0.59265 (12)	0.0515 (7)	
S1S	0.38431 (7)	0.49945 (3)	0.61123 (3)	0.02677 (17)	
C1S	0.2354 (4)	0.48972 (14)	0.57543 (12)	0.0349 (7)	
H1S1	0.256138	0.493617	0.539453	0.052*	
H1S2	0.169147	0.519267	0.585114	0.052*	
H1S3	0.198448	0.450907	0.581931	0.052*	
C2S	0.4379 (4)	0.56766 (14)	0.58597 (14)	0.0403 (8)	
H2S1	0.529001	0.576331	0.597872	0.060*	
H2S2	0.376337	0.598386	0.597128	0.060*	
H2S3	0.437620	0.565697	0.549042	0.060*	

### Atomic displacement parameters (Å^2^)

**Table:**

	*U*^11^	*U*^22^	*U*^33^	*U*^12^	*U*^13^	*U*^23^
C1	0.0184 (13)	0.0231 (13)	0.0142 (11)	0.0022 (11)	0.0009 (10)	−0.0006 (10)
C2	0.0310 (16)	0.0221 (14)	0.0181 (12)	−0.0020 (12)	0.0031 (11)	−0.0027 (11)
C3	0.0286 (16)	0.0250 (14)	0.0219 (13)	−0.0061 (12)	0.0020 (12)	−0.0029 (11)
C4	0.0185 (14)	0.0220 (13)	0.0212 (12)	−0.0029 (11)	0.0024 (11)	−0.0016 (10)
C5	0.0129 (12)	0.0299 (14)	0.0132 (11)	−0.0023 (11)	−0.0023 (10)	0.0002 (11)
C6	0.0155 (13)	0.0275 (14)	0.0133 (11)	−0.0014 (11)	0.0003 (10)	−0.0025 (10)
C7	0.0171 (14)	0.0298 (15)	0.0202 (12)	−0.0018 (11)	−0.0007 (11)	−0.0036 (11)
C8	0.0385 (18)	0.0297 (15)	0.0259 (14)	0.0008 (14)	0.0057 (13)	0.0006 (12)
C9	0.0235 (15)	0.0359 (16)	0.0187 (13)	0.0032 (12)	0.0014 (11)	−0.0049 (12)
C10	0.0186 (14)	0.0261 (13)	0.0141 (11)	−0.0031 (11)	0.0035 (10)	−0.0059 (10)
C11	0.0173 (14)	0.0218 (13)	0.0175 (12)	−0.0058 (10)	0.0011 (10)	−0.0058 (10)
C12	0.0189 (14)	0.0261 (14)	0.0201 (12)	−0.0060 (11)	0.0037 (11)	−0.0013 (11)
C13	0.0291 (16)	0.0198 (13)	0.0199 (13)	−0.0041 (12)	−0.0009 (11)	0.0025 (11)
C14	0.0320 (17)	0.0263 (15)	0.0345 (15)	−0.0097 (13)	0.0002 (13)	0.0065 (13)
C15	0.047 (2)	0.0341 (17)	0.0405 (18)	0.0166 (16)	0.0101 (16)	0.0112 (15)
C16	0.0151 (13)	0.0232 (13)	0.0142 (11)	−0.0033 (11)	0.0024 (10)	0.0016 (10)
C17	0.0156 (13)	0.0233 (13)	0.0131 (11)	0.0002 (10)	0.0027 (10)	0.0004 (10)
C18	0.0176 (13)	0.0227 (13)	0.0190 (12)	−0.0001 (11)	0.0019 (10)	−0.0040 (11)
C19	0.0285 (16)	0.0235 (14)	0.0272 (14)	−0.0029 (12)	−0.0065 (12)	0.0028 (12)
C20	0.052 (2)	0.0257 (16)	0.057 (2)	0.0045 (15)	−0.0255 (19)	0.0011 (16)
C21	0.0241 (15)	0.0276 (14)	0.0194 (13)	−0.0033 (12)	0.0015 (11)	−0.0063 (11)
C22	0.0162 (13)	0.0160 (12)	0.0153 (11)	−0.0039 (10)	−0.0012 (10)	−0.0006 (9)
C23	0.0164 (13)	0.0192 (12)	0.0128 (11)	0.0005 (10)	0.0004 (10)	0.0010 (10)
C24	0.0145 (13)	0.0204 (13)	0.0188 (12)	−0.0013 (10)	0.0002 (10)	−0.0010 (10)
C25	0.0214 (14)	0.0253 (14)	0.0214 (13)	−0.0004 (11)	−0.0034 (11)	−0.0036 (11)
C26	0.0331 (17)	0.0364 (17)	0.0228 (13)	0.0029 (13)	−0.0104 (12)	−0.0004 (12)
C27	0.0201 (14)	0.0217 (13)	0.0239 (13)	−0.0022 (11)	0.0016 (11)	0.0015 (11)
C28	0.0151 (13)	0.0150 (11)	0.0165 (11)	0.0001 (10)	0.0023 (10)	0.0000 (10)
C29	0.0172 (13)	0.0177 (12)	0.0149 (11)	−0.0008 (10)	−0.0008 (10)	0.0000 (10)
C30	0.0209 (14)	0.0181 (13)	0.0172 (12)	0.0014 (11)	0.0007 (10)	−0.0015 (10)
C31	0.0252 (15)	0.0212 (13)	0.0164 (12)	−0.0027 (11)	0.0028 (11)	−0.0032 (10)
C32	0.0321 (17)	0.0267 (15)	0.0286 (14)	0.0006 (13)	0.0070 (12)	−0.0077 (12)
C33	0.0315 (17)	0.0390 (17)	0.0192 (13)	0.0021 (14)	−0.0030 (12)	−0.0053 (12)
C34	0.0139 (12)	0.0215 (13)	0.0105 (10)	−0.0011 (10)	−0.0022 (9)	−0.0016 (9)
C35	0.0141 (13)	0.0186 (12)	0.0144 (11)	−0.0014 (10)	−0.0001 (10)	−0.0012 (10)
C36	0.0190 (13)	0.0175 (13)	0.0212 (13)	−0.0006 (10)	0.0022 (11)	−0.0016 (10)
C37	0.0201 (13)	0.0191 (12)	0.0167 (12)	−0.0007 (10)	0.0039 (10)	−0.0032 (10)
C38	0.0262 (15)	0.0241 (14)	0.0265 (14)	−0.0035 (12)	−0.0027 (12)	−0.0018 (12)
C39	0.0321 (18)	0.0314 (16)	0.0376 (17)	−0.0091 (13)	0.0005 (14)	−0.0080 (14)
C40	0.044 (2)	0.0220 (15)	0.0387 (17)	−0.0102 (14)	0.0149 (15)	−0.0047 (13)
C41	0.0455 (19)	0.0232 (14)	0.0289 (15)	0.0042 (14)	0.0113 (14)	0.0031 (12)
C42	0.0266 (15)	0.0235 (14)	0.0231 (13)	0.0025 (12)	0.0026 (11)	0.0001 (11)
C43	0.0159 (13)	0.0173 (12)	0.0182 (12)	−0.0008 (10)	0.0003 (10)	0.0016 (10)
C44	0.0135 (12)	0.0208 (12)	0.0185 (12)	0.0015 (10)	−0.0002 (10)	−0.0005 (10)
C45	0.0212 (14)	0.0240 (14)	0.0203 (12)	−0.0029 (11)	0.0018 (11)	0.0009 (11)
C46	0.0230 (15)	0.0194 (13)	0.0192 (12)	−0.0056 (11)	0.0003 (11)	0.0034 (10)
C47	0.0240 (15)	0.0257 (14)	0.0277 (14)	−0.0023 (12)	−0.0025 (12)	0.0066 (12)
C48	0.0351 (17)	0.0323 (16)	0.0236 (14)	−0.0093 (14)	−0.0097 (13)	0.0092 (12)
C49	0.049 (2)	0.0266 (15)	0.0142 (12)	−0.0116 (14)	0.0001 (13)	0.0025 (11)
C50	0.0329 (17)	0.0210 (14)	0.0254 (14)	−0.0064 (12)	0.0051 (12)	−0.0025 (11)
C51	0.0228 (14)	0.0202 (13)	0.0216 (13)	−0.0023 (11)	0.0004 (11)	0.0018 (10)
C52	0.0211 (14)	0.0222 (13)	0.0134 (11)	0.0001 (11)	0.0037 (10)	0.0012 (10)
C53	0.0231 (15)	0.0168 (12)	0.0184 (12)	0.0020 (11)	0.0017 (10)	−0.0025 (10)
C54	0.0302 (16)	0.0296 (15)	0.0181 (13)	0.0024 (12)	0.0021 (11)	−0.0073 (11)
C55	0.0319 (16)	0.0339 (16)	0.0149 (12)	0.0011 (13)	0.0033 (11)	−0.0010 (11)
C56	0.0342 (16)	0.0256 (14)	0.0139 (12)	0.0032 (12)	0.0016 (11)	0.0019 (11)
C57	0.0193 (14)	0.0231 (13)	0.0132 (11)	−0.0011 (11)	−0.0013 (10)	−0.0038 (10)
N1	0.0157 (11)	0.0247 (11)	0.0142 (10)	−0.0012 (9)	0.0018 (8)	−0.0009 (9)
N2	0.0129 (11)	0.0337 (13)	0.0120 (10)	−0.0049 (9)	0.0003 (8)	−0.0004 (9)
N3	0.0138 (11)	0.0208 (11)	0.0177 (10)	−0.0011 (9)	0.0007 (8)	−0.0021 (9)
N4	0.0150 (11)	0.0202 (11)	0.0115 (9)	0.0010 (9)	0.0026 (8)	0.0010 (8)
N5	0.0152 (11)	0.0202 (11)	0.0133 (10)	−0.0027 (9)	0.0003 (8)	−0.0005 (8)
N6	0.0169 (11)	0.0196 (11)	0.0150 (10)	0.0003 (9)	0.0036 (8)	0.0012 (9)
N7	0.0166 (11)	0.0200 (11)	0.0148 (10)	0.0028 (9)	0.0018 (8)	0.0008 (8)
N8	0.0169 (11)	0.0205 (11)	0.0159 (10)	0.0011 (9)	−0.0009 (8)	0.0007 (9)
N9	0.0209 (12)	0.0217 (11)	0.0147 (10)	0.0031 (9)	0.0011 (9)	0.0008 (9)
O1	0.0200 (10)	0.0300 (10)	0.0196 (9)	0.0059 (8)	−0.0007 (8)	0.0016 (8)
O2	0.0180 (10)	0.0328 (11)	0.0206 (9)	−0.0071 (8)	0.0035 (8)	−0.0056 (8)
O3	0.0150 (10)	0.0387 (11)	0.0202 (9)	−0.0064 (8)	0.0000 (7)	−0.0022 (8)
O4	0.0149 (10)	0.0278 (10)	0.0245 (9)	−0.0009 (8)	0.0010 (7)	−0.0054 (8)
O5	0.0213 (10)	0.0316 (10)	0.0123 (8)	−0.0013 (8)	0.0016 (7)	0.0030 (7)
O6	0.0219 (10)	0.0198 (9)	0.0185 (9)	−0.0042 (8)	0.0014 (7)	0.0007 (8)
O7	0.0146 (9)	0.0233 (9)	0.0180 (9)	−0.0010 (7)	0.0029 (7)	0.0017 (7)
O8	0.0159 (10)	0.0387 (11)	0.0199 (9)	0.0038 (8)	−0.0009 (8)	−0.0013 (8)
O9	0.0274 (12)	0.0242 (10)	0.0379 (11)	0.0059 (9)	−0.0018 (9)	−0.0037 (9)
O1S	0.0295 (13)	0.0225 (11)	0.102 (2)	0.0109 (10)	0.0132 (14)	0.0067 (13)
S1S	0.0244 (4)	0.0258 (3)	0.0301 (4)	0.0021 (3)	0.0009 (3)	0.0077 (3)
C1S	0.0402 (19)	0.0315 (16)	0.0331 (16)	0.0116 (14)	−0.0101 (14)	−0.0058 (13)
C2S	0.044 (2)	0.0269 (16)	0.050 (2)	0.0049 (15)	0.0154 (17)	0.0083 (15)

### Geometric parameters (Å, º)

**Table:**

C1—N1	1.468 (3)	C29—C30	1.525 (4)
C1—C57	1.533 (4)	C29—H29	1.0000
C1—C2	1.538 (4)	C30—C31	1.540 (3)
C1—H1	1.0000	C30—H30A	0.9900
C2—C3	1.528 (4)	C30—H30B	0.9900
C2—H2A	0.9900	C31—C32	1.525 (4)
C2—H2B	0.9900	C31—C33	1.528 (4)
C3—C4	1.523 (4)	C31—H31	1.0000
C3—H3A	0.9900	C32—H32A	0.9800
C3—H3B	0.9900	C32—H32B	0.9800
C4—N1	1.472 (3)	C32—H32C	0.9800
C4—H4A	0.9900	C33—H33A	0.9800
C4—H4B	0.9900	C33—H33B	0.9800
C5—O2	1.240 (3)	C33—H33C	0.9800
C5—N1	1.340 (3)	C34—O7	1.239 (3)
C5—C6	1.540 (3)	C34—N6	1.345 (3)
C6—N2	1.467 (3)	C34—C35	1.533 (4)
C6—C7	1.535 (4)	C35—N7	1.471 (3)
C6—H6	1.0000	C35—C36	1.527 (4)
C7—C9	1.526 (4)	C35—H35	1.0000
C7—C8	1.531 (4)	C36—C37	1.514 (4)
C7—H7	1.0000	C36—H36A	0.9900
C8—H8A	0.9800	C36—H36B	0.9900
C8—H8B	0.9800	C37—C42	1.386 (4)
C8—H8C	0.9800	C37—C38	1.392 (4)
C9—H9A	0.9800	C38—C39	1.392 (4)
C9—H9B	0.9800	C38—H38	0.9500
C9—H9C	0.9800	C39—C40	1.391 (5)
C10—O3	1.228 (3)	C39—H39	0.9500
C10—N2	1.349 (3)	C40—C41	1.376 (5)
C10—C11	1.556 (4)	C40—H40	0.9500
C11—N3	1.461 (3)	C41—C42	1.387 (4)
C11—C12	1.533 (4)	C41—H41	0.9500
C11—H11	1.0000	C42—H42	0.9500
C12—C13	1.525 (4)	C43—O8	1.230 (3)
C12—H12A	0.9900	C43—N7	1.355 (3)
C12—H12B	0.9900	C43—C44	1.531 (4)
C13—C15	1.524 (4)	C44—N8	1.457 (3)
C13—C14	1.528 (4)	C44—C45	1.536 (4)
C13—H13	1.0000	C44—H44	1.0000
C14—H14A	0.9800	C45—C46	1.514 (4)
C14—H14B	0.9800	C45—H45A	0.9900
C14—H14C	0.9800	C45—H45B	0.9900
C15—H15A	0.9800	C46—C47	1.388 (4)
C15—H15B	0.9800	C46—C51	1.399 (4)
C15—H15C	0.9800	C47—C48	1.397 (4)
C16—O4	1.230 (3)	C47—H47	0.9500
C16—N3	1.352 (3)	C48—C49	1.383 (5)
C16—C17	1.524 (4)	C48—H48	0.9500
C17—N4	1.455 (3)	C49—C50	1.390 (5)
C17—C18	1.536 (4)	C49—H49	0.9500
C17—H17	1.0000	C50—C51	1.395 (4)
C18—C19	1.529 (4)	C50—H50	0.9500
C18—C21	1.532 (4)	C51—H51	0.9500
C18—H18	1.0000	C52—O9	1.234 (3)
C19—C20	1.521 (4)	C52—N8	1.346 (3)
C19—H19A	0.9900	C52—C53	1.527 (4)
C19—H19B	0.9900	C53—N9	1.466 (3)
C20—H20A	0.9800	C53—C54	1.544 (4)
C20—H20B	0.9800	C53—H53	1.0000
C20—H20C	0.9800	C54—C55	1.518 (4)
C21—H21A	0.9800	C54—H54A	0.9900
C21—H21B	0.9800	C54—H54B	0.9900
C21—H21C	0.9800	C55—C56	1.521 (4)
C22—O5	1.222 (3)	C55—H55A	0.9900
C22—N4	1.358 (3)	C55—H55B	0.9900
C22—C23	1.528 (4)	C56—N9	1.483 (3)
C23—N5	1.467 (3)	C56—H56A	0.9900
C23—C24	1.548 (4)	C56—H56B	0.9900
C23—H23	1.0000	C57—O1	1.226 (3)
C24—C27	1.533 (4)	C57—N9	1.359 (3)
C24—C25	1.535 (4)	N2—H2N	0.8800
C24—H24	1.0000	N3—H3N	0.8800
C25—C26	1.518 (4)	N4—H4N	0.8800
C25—H25A	0.9900	N5—H5N	0.8800
C25—H25B	0.9900	N6—H6N	0.8800
C26—H26A	0.9800	N8—H8N	0.8800
C26—H26B	0.9800	O1S—S1S	1.486 (2)
C26—H26C	0.9800	S1S—C1S	1.773 (3)
C27—H27A	0.9800	S1S—C2S	1.786 (3)
C27—H27B	0.9800	C1S—H1S1	0.9800
C27—H27C	0.9800	C1S—H1S2	0.9800
C28—O6	1.237 (3)	C1S—H1S3	0.9800
C28—N5	1.346 (3)	C2S—H2S1	0.9800
C28—C29	1.540 (3)	C2S—H2S2	0.9800
C29—N6	1.455 (3)	C2S—H2S3	0.9800
			
N1—C1—C57	110.1 (2)	C29—C30—H30A	108.3
N1—C1—C2	102.9 (2)	C31—C30—H30A	108.3
C57—C1—C2	110.7 (2)	C29—C30—H30B	108.3
N1—C1—H1	111.0	C31—C30—H30B	108.3
C57—C1—H1	111.0	H30A—C30—H30B	107.4
C2—C1—H1	111.0	C32—C31—C33	110.6 (2)
C3—C2—C1	103.2 (2)	C32—C31—C30	108.2 (2)
C3—C2—H2A	111.1	C33—C31—C30	113.1 (2)
C1—C2—H2A	111.1	C32—C31—H31	108.3
C3—C2—H2B	111.1	C33—C31—H31	108.3
C1—C2—H2B	111.1	C30—C31—H31	108.3
H2A—C2—H2B	109.1	C31—C32—H32A	109.5
C4—C3—C2	103.5 (2)	C31—C32—H32B	109.5
C4—C3—H3A	111.1	H32A—C32—H32B	109.5
C2—C3—H3A	111.1	C31—C32—H32C	109.5
C4—C3—H3B	111.1	H32A—C32—H32C	109.5
C2—C3—H3B	111.1	H32B—C32—H32C	109.5
H3A—C3—H3B	109.0	C31—C33—H33A	109.5
N1—C4—C3	103.3 (2)	C31—C33—H33B	109.5
N1—C4—H4A	111.1	H33A—C33—H33B	109.5
C3—C4—H4A	111.1	C31—C33—H33C	109.5
N1—C4—H4B	111.1	H33A—C33—H33C	109.5
C3—C4—H4B	111.1	H33B—C33—H33C	109.5
H4A—C4—H4B	109.1	O7—C34—N6	121.2 (2)
O2—C5—N1	121.4 (2)	O7—C34—C35	122.9 (2)
O2—C5—C6	122.8 (2)	N6—C34—C35	115.9 (2)
N1—C5—C6	115.7 (2)	N7—C35—C36	111.8 (2)
N2—C6—C7	113.2 (2)	N7—C35—C34	110.8 (2)
N2—C6—C5	105.9 (2)	C36—C35—C34	111.7 (2)
C7—C6—C5	113.3 (2)	N7—C35—H35	107.4
N2—C6—H6	108.1	C36—C35—H35	107.4
C7—C6—H6	108.1	C34—C35—H35	107.4
C5—C6—H6	108.1	C37—C36—C35	114.4 (2)
C9—C7—C8	109.8 (2)	C37—C36—H36A	108.7
C9—C7—C6	109.7 (2)	C35—C36—H36A	108.7
C8—C7—C6	110.4 (2)	C37—C36—H36B	108.7
C9—C7—H7	109.0	C35—C36—H36B	108.7
C8—C7—H7	109.0	H36A—C36—H36B	107.6
C6—C7—H7	109.0	C42—C37—C38	118.5 (3)
C7—C8—H8A	109.5	C42—C37—C36	121.2 (2)
C7—C8—H8B	109.5	C38—C37—C36	120.2 (2)
H8A—C8—H8B	109.5	C37—C38—C39	120.4 (3)
C7—C8—H8C	109.5	C37—C38—H38	119.8
H8A—C8—H8C	109.5	C39—C38—H38	119.8
H8B—C8—H8C	109.5	C40—C39—C38	120.1 (3)
C7—C9—H9A	109.5	C40—C39—H39	120.0
C7—C9—H9B	109.5	C38—C39—H39	120.0
H9A—C9—H9B	109.5	C41—C40—C39	119.7 (3)
C7—C9—H9C	109.5	C41—C40—H40	120.1
H9A—C9—H9C	109.5	C39—C40—H40	120.1
H9B—C9—H9C	109.5	C40—C41—C42	120.0 (3)
O3—C10—N2	123.6 (3)	C40—C41—H41	120.0
O3—C10—C11	121.7 (2)	C42—C41—H41	120.0
N2—C10—C11	114.5 (2)	C37—C42—C41	121.3 (3)
N3—C11—C12	114.8 (2)	C37—C42—H42	119.4
N3—C11—C10	111.0 (2)	C41—C42—H42	119.4
C12—C11—C10	112.2 (2)	O8—C43—N7	122.2 (2)
N3—C11—H11	106.1	O8—C43—C44	119.8 (2)
C12—C11—H11	106.1	N7—C43—C44	118.0 (2)
C10—C11—H11	106.1	N8—C44—C43	113.2 (2)
C13—C12—C11	113.1 (2)	N8—C44—C45	112.2 (2)
C13—C12—H12A	109.0	C43—C44—C45	110.1 (2)
C11—C12—H12A	109.0	N8—C44—H44	107.0
C13—C12—H12B	109.0	C43—C44—H44	107.0
C11—C12—H12B	109.0	C45—C44—H44	107.0
H12A—C12—H12B	107.8	C46—C45—C44	112.3 (2)
C15—C13—C12	111.2 (3)	C46—C45—H45A	109.1
C15—C13—C14	110.4 (2)	C44—C45—H45A	109.1
C12—C13—C14	110.5 (2)	C46—C45—H45B	109.1
C15—C13—H13	108.2	C44—C45—H45B	109.1
C12—C13—H13	108.2	H45A—C45—H45B	107.9
C14—C13—H13	108.2	C47—C46—C51	119.0 (3)
C13—C14—H14A	109.5	C47—C46—C45	121.2 (3)
C13—C14—H14B	109.5	C51—C46—C45	119.8 (2)
H14A—C14—H14B	109.5	C46—C47—C48	120.4 (3)
C13—C14—H14C	109.5	C46—C47—H47	119.8
H14A—C14—H14C	109.5	C48—C47—H47	119.8
H14B—C14—H14C	109.5	C49—C48—C47	120.5 (3)
C13—C15—H15A	109.5	C49—C48—H48	119.8
C13—C15—H15B	109.5	C47—C48—H48	119.8
H15A—C15—H15B	109.5	C48—C49—C50	119.6 (3)
C13—C15—H15C	109.5	C48—C49—H49	120.2
H15A—C15—H15C	109.5	C50—C49—H49	120.2
H15B—C15—H15C	109.5	C49—C50—C51	120.2 (3)
O4—C16—N3	122.1 (2)	C49—C50—H50	119.9
O4—C16—C17	121.5 (2)	C51—C50—H50	119.9
N3—C16—C17	116.4 (2)	C50—C51—C46	120.3 (3)
N4—C17—C16	111.5 (2)	C50—C51—H51	119.8
N4—C17—C18	112.9 (2)	C46—C51—H51	119.8
C16—C17—C18	111.6 (2)	O9—C52—N8	124.0 (3)
N4—C17—H17	106.8	O9—C52—C53	118.0 (2)
C16—C17—H17	106.8	N8—C52—C53	118.0 (2)
C18—C17—H17	106.8	N9—C53—C52	115.9 (2)
C19—C18—C21	111.6 (2)	N9—C53—C54	102.3 (2)
C19—C18—C17	112.2 (2)	C52—C53—C54	111.0 (2)
C21—C18—C17	110.1 (2)	N9—C53—H53	109.1
C19—C18—H18	107.6	C52—C53—H53	109.1
C21—C18—H18	107.6	C54—C53—H53	109.1
C17—C18—H18	107.6	C55—C54—C53	103.2 (2)
C20—C19—C18	113.0 (3)	C55—C54—H54A	111.1
C20—C19—H19A	109.0	C53—C54—H54A	111.1
C18—C19—H19A	109.0	C55—C54—H54B	111.1
C20—C19—H19B	109.0	C53—C54—H54B	111.1
C18—C19—H19B	109.0	H54A—C54—H54B	109.1
H19A—C19—H19B	107.8	C54—C55—C56	103.8 (2)
C19—C20—H20A	109.5	C54—C55—H55A	111.0
C19—C20—H20B	109.5	C56—C55—H55A	111.0
H20A—C20—H20B	109.5	C54—C55—H55B	111.0
C19—C20—H20C	109.5	C56—C55—H55B	111.0
H20A—C20—H20C	109.5	H55A—C55—H55B	109.0
H20B—C20—H20C	109.5	N9—C56—C55	103.0 (2)
C18—C21—H21A	109.5	N9—C56—H56A	111.2
C18—C21—H21B	109.5	C55—C56—H56A	111.2
H21A—C21—H21B	109.5	N9—C56—H56B	111.2
C18—C21—H21C	109.5	C55—C56—H56B	111.2
H21A—C21—H21C	109.5	H56A—C56—H56B	109.1
H21B—C21—H21C	109.5	O1—C57—N9	121.8 (2)
O5—C22—N4	123.0 (2)	O1—C57—C1	121.3 (2)
O5—C22—C23	119.6 (2)	N9—C57—C1	116.8 (2)
N4—C22—C23	117.3 (2)	C5—N1—C1	120.3 (2)
N5—C23—C22	114.0 (2)	C5—N1—C4	126.9 (2)
N5—C23—C24	107.5 (2)	C1—N1—C4	112.5 (2)
C22—C23—C24	110.9 (2)	C10—N2—C6	123.3 (2)
N5—C23—H23	108.1	C10—N2—H2N	118.3
C22—C23—H23	108.1	C6—N2—H2N	118.3
C24—C23—H23	108.1	C16—N3—C11	121.0 (2)
C27—C24—C25	109.9 (2)	C16—N3—H3N	119.5
C27—C24—C23	113.2 (2)	C11—N3—H3N	119.5
C25—C24—C23	113.4 (2)	C22—N4—C17	120.7 (2)
C27—C24—H24	106.6	C22—N4—H4N	119.6
C25—C24—H24	106.6	C17—N4—H4N	119.6
C23—C24—H24	106.6	C28—N5—C23	125.1 (2)
C26—C25—C24	114.7 (2)	C28—N5—H5N	117.5
C26—C25—H25A	108.6	C23—N5—H5N	117.5
C24—C25—H25A	108.6	C34—N6—C29	120.3 (2)
C26—C25—H25B	108.6	C34—N6—H6N	119.9
C24—C25—H25B	108.6	C29—N6—H6N	119.9
H25A—C25—H25B	107.6	C43—N7—C35	120.2 (2)
C25—C26—H26A	109.5	C52—N8—C44	120.7 (2)
C25—C26—H26B	109.5	C52—N8—H8N	119.7
H26A—C26—H26B	109.5	C44—N8—H8N	119.7
C25—C26—H26C	109.5	C57—N9—C53	125.9 (2)
H26A—C26—H26C	109.5	C57—N9—C56	118.9 (2)
H26B—C26—H26C	109.5	C53—N9—C56	112.4 (2)
C24—C27—H27A	109.5	O1S—S1S—C1S	106.62 (16)
C24—C27—H27B	109.5	O1S—S1S—C2S	105.92 (15)
H27A—C27—H27B	109.5	C1S—S1S—C2S	99.15 (18)
C24—C27—H27C	109.5	S1S—C1S—H1S1	109.5
H27A—C27—H27C	109.5	S1S—C1S—H1S2	109.5
H27B—C27—H27C	109.5	H1S1—C1S—H1S2	109.5
O6—C28—N5	124.0 (2)	S1S—C1S—H1S3	109.5
O6—C28—C29	120.6 (2)	H1S1—C1S—H1S3	109.5
N5—C28—C29	115.4 (2)	H1S2—C1S—H1S3	109.5
N6—C29—C30	111.8 (2)	S1S—C2S—H2S1	109.5
N6—C29—C28	111.4 (2)	S1S—C2S—H2S2	109.5
C30—C29—C28	109.9 (2)	H2S1—C2S—H2S2	109.5
N6—C29—H29	107.8	S1S—C2S—H2S3	109.5
C30—C29—H29	107.8	H2S1—C2S—H2S3	109.5
C28—C29—H29	107.8	H2S2—C2S—H2S3	109.5
C29—C30—C31	115.9 (2)		
			
N1—C1—C2—C3	31.1 (3)	C44—C45—C46—C47	107.3 (3)
C57—C1—C2—C3	−86.5 (3)	C44—C45—C46—C51	−72.4 (3)
C1—C2—C3—C4	−38.7 (3)	C51—C46—C47—C48	−0.2 (4)
C2—C3—C4—N1	30.8 (3)	C45—C46—C47—C48	−179.9 (3)
O2—C5—C6—N2	−101.8 (3)	C46—C47—C48—C49	−0.5 (4)
N1—C5—C6—N2	75.2 (3)	C47—C48—C49—C50	0.9 (4)
O2—C5—C6—C7	22.8 (4)	C48—C49—C50—C51	−0.7 (4)
N1—C5—C6—C7	−160.2 (2)	C49—C50—C51—C46	0.1 (4)
N2—C6—C7—C9	−58.2 (3)	C47—C46—C51—C50	0.3 (4)
C5—C6—C7—C9	−178.7 (2)	C45—C46—C51—C50	−179.9 (3)
N2—C6—C7—C8	−179.3 (2)	O9—C52—C53—N9	170.8 (2)
C5—C6—C7—C8	60.1 (3)	N8—C52—C53—N9	−10.3 (3)
O3—C10—C11—N3	−143.9 (2)	O9—C52—C53—C54	−73.2 (3)
N2—C10—C11—N3	40.5 (3)	N8—C52—C53—C54	105.7 (3)
O3—C10—C11—C12	−14.1 (3)	N9—C53—C54—C55	33.0 (3)
N2—C10—C11—C12	170.4 (2)	C52—C53—C54—C55	−91.1 (3)
N3—C11—C12—C13	−135.4 (2)	C53—C54—C55—C56	−39.7 (3)
C10—C11—C12—C13	96.7 (3)	C54—C55—C56—N9	30.2 (3)
C11—C12—C13—C15	80.0 (3)	N1—C1—C57—O1	−20.4 (3)
C11—C12—C13—C14	−157.0 (2)	C2—C1—C57—O1	92.7 (3)
O4—C16—C17—N4	−157.3 (2)	N1—C1—C57—N9	161.9 (2)
N3—C16—C17—N4	23.5 (3)	C2—C1—C57—N9	−85.1 (3)
O4—C16—C17—C18	−30.0 (3)	O2—C5—N1—C1	−2.1 (4)
N3—C16—C17—C18	150.8 (2)	C6—C5—N1—C1	−179.2 (2)
N4—C17—C18—C19	60.2 (3)	O2—C5—N1—C4	−175.9 (2)
C16—C17—C18—C19	−66.3 (3)	C6—C5—N1—C4	7.0 (4)
N4—C17—C18—C21	−64.7 (3)	C57—C1—N1—C5	−68.9 (3)
C16—C17—C18—C21	168.7 (2)	C2—C1—N1—C5	173.1 (2)
C21—C18—C19—C20	−78.8 (3)	C57—C1—N1—C4	105.7 (2)
C17—C18—C19—C20	157.0 (3)	C2—C1—N1—C4	−12.3 (3)
O5—C22—C23—N5	155.1 (2)	C3—C4—N1—C5	162.6 (2)
N4—C22—C23—N5	−28.6 (3)	C3—C4—N1—C1	−11.6 (3)
O5—C22—C23—C24	−83.5 (3)	O3—C10—N2—C6	−2.3 (4)
N4—C22—C23—C24	92.9 (3)	C11—C10—N2—C6	173.1 (2)
N5—C23—C24—C27	58.5 (3)	C7—C6—N2—C10	109.7 (3)
C22—C23—C24—C27	−66.7 (3)	C5—C6—N2—C10	−125.5 (3)
N5—C23—C24—C25	−175.3 (2)	O4—C16—N3—C11	−3.3 (4)
C22—C23—C24—C25	59.4 (3)	C17—C16—N3—C11	175.9 (2)
C27—C24—C25—C26	−175.4 (2)	C12—C11—N3—C16	−69.8 (3)
C23—C24—C25—C26	56.7 (3)	C10—C11—N3—C16	58.7 (3)
O6—C28—C29—N6	145.9 (2)	O5—C22—N4—C17	−4.1 (4)
N5—C28—C29—N6	−33.8 (3)	C23—C22—N4—C17	179.7 (2)
O6—C28—C29—C30	−89.6 (3)	C16—C17—N4—C22	−124.2 (2)
N5—C28—C29—C30	90.7 (3)	C18—C17—N4—C22	109.2 (3)
N6—C29—C30—C31	−56.9 (3)	O6—C28—N5—C23	−0.4 (4)
C28—C29—C30—C31	178.8 (2)	C29—C28—N5—C23	179.3 (2)
C29—C30—C31—C32	−174.6 (2)	C22—C23—N5—C28	−52.7 (3)
C29—C30—C31—C33	−51.8 (3)	C24—C23—N5—C28	−176.0 (2)
O7—C34—C35—N7	−122.8 (2)	O7—C34—N6—C29	2.4 (4)
N6—C34—C35—N7	59.4 (3)	C35—C34—N6—C29	−179.9 (2)
O7—C34—C35—C36	2.5 (3)	C30—C29—N6—C34	176.5 (2)
N6—C34—C35—C36	−175.2 (2)	C28—C29—N6—C34	−60.1 (3)
N7—C35—C36—C37	−80.8 (3)	O8—C43—N7—C35	−0.1 (4)
C34—C35—C36—C37	154.4 (2)	C44—C43—N7—C35	−178.8 (2)
C35—C36—C37—C42	119.6 (3)	C36—C35—N7—C43	149.9 (2)
C35—C36—C37—C38	−62.9 (3)	C34—C35—N7—C43	−84.8 (3)
C42—C37—C38—C39	−1.2 (4)	O9—C52—N8—C44	1.5 (4)
C36—C37—C38—C39	−178.8 (3)	C53—C52—N8—C44	−177.3 (2)
C37—C38—C39—C40	0.7 (5)	C43—C44—N8—C52	−103.3 (3)
C38—C39—C40—C41	0.1 (5)	C45—C44—N8—C52	131.3 (2)
C39—C40—C41—C42	−0.3 (5)	O1—C57—N9—C53	−166.3 (2)
C38—C37—C42—C41	1.0 (4)	C1—C57—N9—C53	11.4 (4)
C36—C37—C42—C41	178.6 (3)	O1—C57—N9—C56	−7.0 (4)
C40—C41—C42—C37	−0.3 (4)	C1—C57—N9—C56	170.8 (2)
O8—C43—C44—N8	140.3 (2)	C52—C53—N9—C57	−93.4 (3)
N7—C43—C44—N8	−40.9 (3)	C54—C53—N9—C57	145.8 (3)
O8—C43—C44—C45	−93.2 (3)	C52—C53—N9—C56	106.1 (3)
N7—C43—C44—C45	85.6 (3)	C54—C53—N9—C56	−14.7 (3)
N8—C44—C45—C46	−59.0 (3)	C55—C56—N9—C57	−171.5 (2)
C43—C44—C45—C46	174.0 (2)	C55—C56—N9—C53	−9.5 (3)

### Hydrogen-bond geometry (Å, º)

**Table:**

*D*—H···*A*	*D*—H	H···*A*	*D*···*A*	*D*—H···*A*
N2—H2*N*···O7	0.88	2.12	2.970 (3)	162
N3—H3*N*···O6	0.88	2.16	3.023 (3)	166
N4—H4*N*···O7	0.88	2.29	3.117 (3)	157
N5—H5*N*···O3^i^	0.88	2.38	3.094 (3)	139
N6—H6*N*···O8	0.88	2.16	2.926 (3)	145
N8—H8*N*···O1*S*	0.88	2.05	2.800 (3)	150

Symmetry code: (i) *x*+1, *y*, *z*.
